# Conformational dynamics of the nucleotide binding domains and the power stroke of a heterodimeric ABC transporter

**DOI:** 10.7554/eLife.02740

**Published:** 2014-05-16

**Authors:** Smriti Mishra, Brandy Verhalen, Richard A Stein, Po-Chao Wen, Emad Tajkhorshid, Hassane S Mchaourab

**Affiliations:** 1 Department of Molecular Physiology and Biophysics, Vanderbilt University, Nashville, United States; 2 Department of Biochemistry, College of Medicine, University of Illinois, Urbana, United States; 3 Center for Biophysics and Computational Biology, University of Illinois, Urbana, United States; 4 The Beckman Institute for Advanced Science and Technology, University of Illinois, Urbana, Unites States; Goethe University, Germany

**Keywords:** ABC transporters, double electron electron resonance (DEER), ABC heterodimers, none

## Abstract

Multidrug ATP binding cassette (ABC) exporters are ubiquitous ABC transporters that extrude cytotoxic molecules across cell membranes. Despite recent progress in structure determination of these transporters, the conformational motion that transduces the energy of ATP hydrolysis to the work of substrate translocation remains undefined. Here, we have investigated the conformational cycle of BmrCD, a representative of the heterodimer family of ABC exporters that have an intrinsically impaired nucleotide binding site. We measured distances between pairs of spin labels monitoring the movement of the nucleotide binding (NBD) and transmembrane domains (TMD). The results expose previously unobserved structural intermediates of the NBDs arising from asymmetric configuration of catalytically inequivalent nucleotide binding sites. The two-state transition of the TMD, from an inward- to an outward-facing conformation, is driven exclusively by ATP hydrolysis. These findings provide direct evidence of divergence in the mechanism of ABC exporters.

**DOI:** http://dx.doi.org/10.7554/eLife.02740.001

## eLife digest

Cells are surrounded by a membrane that acts like a barrier to many molecules. This membrane either stops molecules from entering or exiting the cell, or at least slows their movement. However, it is important that cells can remove some molecules, such as toxins, and that nutrients and certain other molecules can get into cells. As such, cells rely on ‘transporter’ proteins embedded within the membrane to move these molecules through the membrane.

Transporters called ‘Multidrug ABC exporters’ are found in almost all living things, and use the energy released by breaking down molecules of adenosine triphosphate (ATP for short) to pump toxins out of cells. Although the three-dimensional shapes of many transporters are known, it is not clear how the energy released from ATP molecules allows the transporter to move a toxin from one side of the membrane to the other.

Here, Mishra et al. have looked at how the shape of an ABC exporter from a bacterium called *Bacillus subtilis* changes as it interacts with ATP. Most bacterial ABC exporters are made from two copies of the same protein, but the *B. subtilis* exporter is made from two slightly different proteins, one of which is less able to bind to and break down ATP. Mishra et al. found that those parts of the two proteins that bind to ATP can adopt a range of different shapes that had not been seen before. Moreover, the parts of the proteins that extend across the cell membrane face into the cell when the ATP binds, and switch to face out of the cell when the ATP is broken down. This movement of the proteins would allow toxic molecules inside the cell to enter the exporter, and then be pushed to the outside of the cell.

The findings of Mishra et al. show that not all ABC exporters work by the same mechanism. Future work could extend this new understanding to multidrug ABC transporters from humans, which remove waste and harmful molecules from our cells and have been implicated in resistance to chemotherapy in cancer cells.

**DOI:** http://dx.doi.org/10.7554/eLife.02740.002

## Introduction

ATP binding cassette (ABC) transporters harness the energy of ATP to traffic a wide spectrum of molecules across cell membranes. In prokaryotes, ABC importers drive accumulation of nutrients in the cytoplasm against their concentration gradients while ABC exporters remove toxic substrates out of the cytoplasm and may function as flippases of lipids (Higgins and Linton, 2004; Rees et al., 2009; Sharom, 2011; George and Jones, 2012). Mammalian ABC transporters, such as P-glycoprotein (Pgp) and cystic fibrosis transmembrane conductance regulator (CFTR), are exclusively of the exporter class, play critical physiological roles and are associated with disease (Higgins and Linton, 2004). Importers and exporters share a modular molecular architecture featuring two nucleotide binding domains (NBDs or ATP binding cassettes) that turnover ATP and two transmembrane domains (TMDs) that are presumed to form a translocation pathway across the bilayer. The four modules of ABC transporters can be encoded by separate genes and assembled as homo- or hetero-dimers, or expressed as a single polypeptide chain (Higgins and Linton, 2004; Rees et al., 2009).

Mapping the conformational motion that transduces the energy of ATP binding and hydrolysis in the NBDs to the mechanical work of substrate translocation in the TMDs is central to understanding the mechanism of ABC transporters. Crystallographic snapshots of ABC importers have revealed inward- and outward-facing states (Locher et al., 2002; Hollenstein et al., 2007; Oldham et al., 2008; Korkhov et al., 2012) in the nomenclature of Jardetzky's alternating access model (Jardetzky, 1966). Determined in the presence of substrates, substrate binding proteins and/or nucleotides, these structures were cast as representing catalytic intermediates in the ATP binding and hydrolysis cycle. In contrast, the proposed structural mechanism of ABC exporters is less elaborate invoking two states captured by crystallography: Inward-facing devoid of substrates and/or nucleotides (referred to as apo) (Ward et al., 2007; Aller et al., 2009; Jin et al., 2012) and outward-facing with bound nucleotides (Dawson and Locher, 2006, 2007; Ward et al., 2007). While these structures highlight the possible range of conformational motion, there is no consensus regarding the suite of conformational steps that couple ATP hydrolysis to substrate translocation (George and Jones, 2012).

The quest for a unified mechanism of transport by ABC exporters has been hampered by seemingly conflicting structural and biochemical models. Inward-facing structures of the bacterial homodimer MsbA (Ward et al., 2007) and eukaryotic Pgp (Aller et al., 2009; Jin et al., 2012) have inverted V-shapes wherein the two leaflets of the transporters are separated by a large chamber open to the cytoplasm and the bilayer. In these nucleotide and substrate-free structures, the two NBDs are disengaged and separated by 10–50 Å. In contrast, nucleotide-bound, outward-facing structures of MsbA (Ward et al., 2007) and its homolog Sav1866 (Dawson and Locher, 2006, 2007) have the two NBDs in close contact, bringing together the ABC signature motif from one subunit and the Walker A and Walker B sequences from the other subunit to form the nucleotide binding sites (NBSs). Inward- and outward-facing exporter structures were interpreted to imply that transport entails cycles of association and dissociation of the NBDs powered by ATP binding and hydrolysis. In this model, substrate partitions into the large chamber cradled by the two TMDs and is pushed along to the outer membrane leaflet by ATP-induced alternating access of the chamber. However, central elements of this model are considered inconsistent with mechanistic studies, primarily of the mammalian ABC exporter Pgp, implying catalytic asymmetry between the two NBSs and constant contact between the NBDs during transport (Tombline and Senior, 2005; Siarheyeva et al., 2010; George and Jones, 2012). Furthermore, it would appear that an apo state, devoid of nucleotides, is unlikely to be populated or is transient under cellular ATP concentrations which are typically an order of magnitude above the *K*_m_ of ATP. The ‘alternating site hydrolysis’ model posits that the two NBSs turnover ATP in an alternating manner constantly holding an intact ATP molecule in one of the NBSs (Tombline and Senior, 2005). The conjecture that the large inward-facing cavity observed in the apo structures is required to accommodate the large substrates of exporters has also been challenged (George and Jones, 2012) by the observation of smaller openings in the outward-facing conformations of MsbA and Sav1866 (Dawson and Locher, 2006, 2007; Ward et al., 2007).

Further confounding the structural perspective, a recent structure of an ABC heterodimer TM287/288 from the hyperthermophile *Thermotoga maritima* offered the first view of an ABC exporter in an inward-facing conformation where the two NBDs are partially engaged (Hohl et al., 2012). Unlike MsbA, Sav1866 and Pgp, TM287/288 is a heterodimeric ABC transporter with non-canonical sequences in the Walker B and switch motifs of one subunit resulting in a catalytically impaired NBS. In the TM287/288 structure, the impaired NBS, also referred to as the degenerate NBS, is bridged by an AMP-PNP molecule while the intact NBS, referred to as the consensus NBS, is more open creating an asymmetric NBD interface. Despite the partially engaged NBDs, the TMDs still form a chamber open to partitioning of substrates from the cytoplasm but does not appear to be accessible from the bilayer (Hohl et al., 2012). This structure is consistent with biochemical studies of bacterial ABC heterodimers (Lubelski et al., 2006; Zutz et al., 2011) that suggest catalytic asymmetry between the NBSs. However, while the TM287/288 structure provides a structural basis for asymmetric nucleotide binding, it does not elucidate the mechanistic role of such an intermediate in the transport cycle in the presence of substrate and under conditions of ATP turnover. Importantly, the broader question of how the mechanism of homodimers and heterodimers differ as a consequence of impairment of an NBS remains unanswered. In addition to their importance to understanding the fundamental mechanism of transport, addressing these questions provides insight into the role of catalytic asymmetry in mammalian ABC transporters such as CFTR and TAP1/2, which play fundamental physiological roles and have been directly associated with diseases (Abele and Tampé, 2004; Aleksandrov et al., 2007).

Here, we have investigated the conformational changes associated with specific steps in the substrate-coupled ATPase cycle of an ABC heterodimeric transporter, BmrCD from *Bacillus subtilis* (Torres et al., 2009; Galián et al., 2011). Similar to other ABC heterodimers, sequence modifications in the consensus motifs of the NBDs, including the replacement of the Walker B catalytic glutamate by an aspartate in BmrD, suggest that ATP turnover is impaired. *BmrC* and *BmrD* genes are upregulated in the response of *B. subtilis* to antibiotics exposure and BmrCD confers multidrug transport activity on inside-out *Escherichia coli* membrane vesicles (Torres et al., 2009) and in reconstituted giant unilamellar vesicles (Dezi et al., 2013). Spin label pairs were introduced at strategic locations to monitor the movement of the NBDs and TMDs by Double electron–electron resonance spectroscopy (DEER) (Jeschke and Polyhach, 2007; Mchaourab et al., 2011). The experimental design reconstructs the conformational dynamics of the transporter by comparing distance distributions obtained under turnover conditions with those measured in trapped catalytic intermediates. Our results reveal structural asymmetry at the NBSs presumably reflecting asymmetric binding and hydrolysis of ATP. The ATPase cycle proceeds through multiple conformations of the NBD dimer while the TMDs undergo an inward- to outward-facing transition powered exclusively by ATP hydrolysis. These are distinct mechanistic steps compared to ABC homodimers where a two-state association/dissociation cycle of the NBD is tightly coupled to the transition of the TMD from inward- to outward-facing conformations with ATP binding providing the power stroke (Dong et al., 2005; Zou and Mchaourab, 2009). To our knowledge, this is the first study to define the structural dynamic consequences of the selective impairment of an NBS and to uncover divergence in the conformational cycles within the ABC exporter superfamily.

## Results

**Figure 1.**
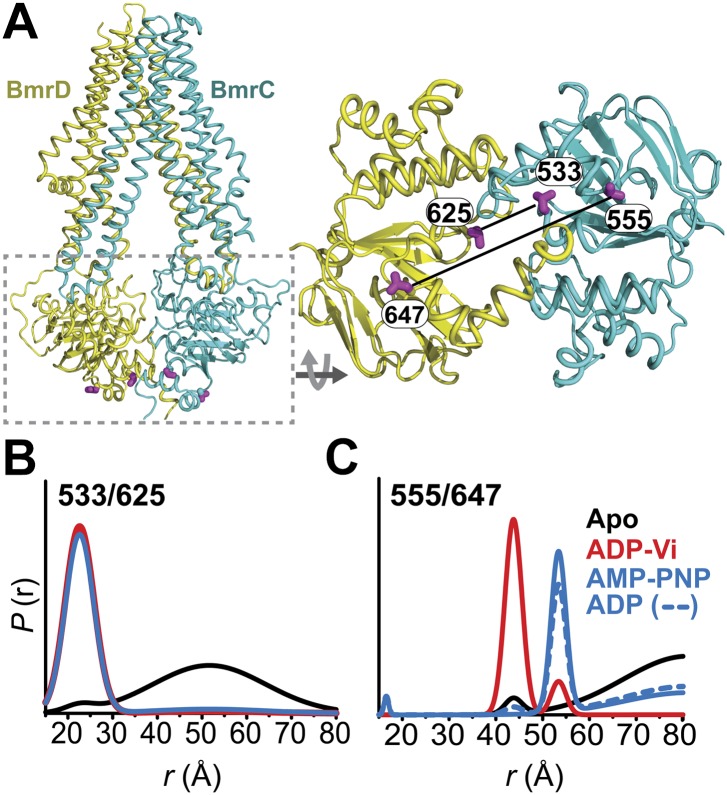
Conformational states of BmrCD NBDs. (**A**) Ribbon representation of BmrCD homology model showing the spin label pairs at symmetric sites across the NBD dimer interface (the transporter is rotated 90° and NBD interface is viewed from cytoplasm). (**B** and **C**) Distance distributions revealing conformational changes upon ADP and AMP-PNP binding (blue traces) and ATP hydrolysis (red traces). ATP binding mimicked by AMP-PNP induces the formation of an intermediate state distinct from that stabilized by ATP hydrolysis. **DOI:** http://dx.doi.org/10.7554/eLife.02740.003

To monitor conformational changes in BmrCD as it cycles between different states driven by substrate binding and ATP turnover, we introduced pairs of spin labels, consisting of one spin label in BmrC and one spin label in BmrD, at surface-exposed sites in the NBDs and TMDs. The native cysteines of BmrD were replaced by alanines to yield a cysteine-free construct. To guide in the selection of residues for spin labeling, a homology model of BmrCD was built using the crystal structure of AMP-PNP-bound TM287/288 (Hohl et al., 2012) as a template (Figure 1A, Figure 1—figure supplement 1). A subset of the spin label pairs was designed to be structurally similar to MsbA pairs previously demonstrated to ‘fingerprint’ the transition between inward- and outward-facing conformations (Borbat et al., 2007; Zou et al., 2009). We verified that cysteine-free and spin-labeled BmrCD mutants hydrolyze ATP in dodecyl maltoside (DDM) micelles and in lipid bilayers (Figure 1—figure supplement 2, Figure 4—figure supplement 2). Importanly, the rates of ATP turnover are stimulated by the transport substrate Hoechst33342 (hereafter referred as Hoechst) except for the 533/625 pair. Therefore, this pair will be interpreted only in the context of the overall data set describing the movement of the NBDs as a consequence of ATP hydrolysis.

**Figure 1—figure supplement 1.**
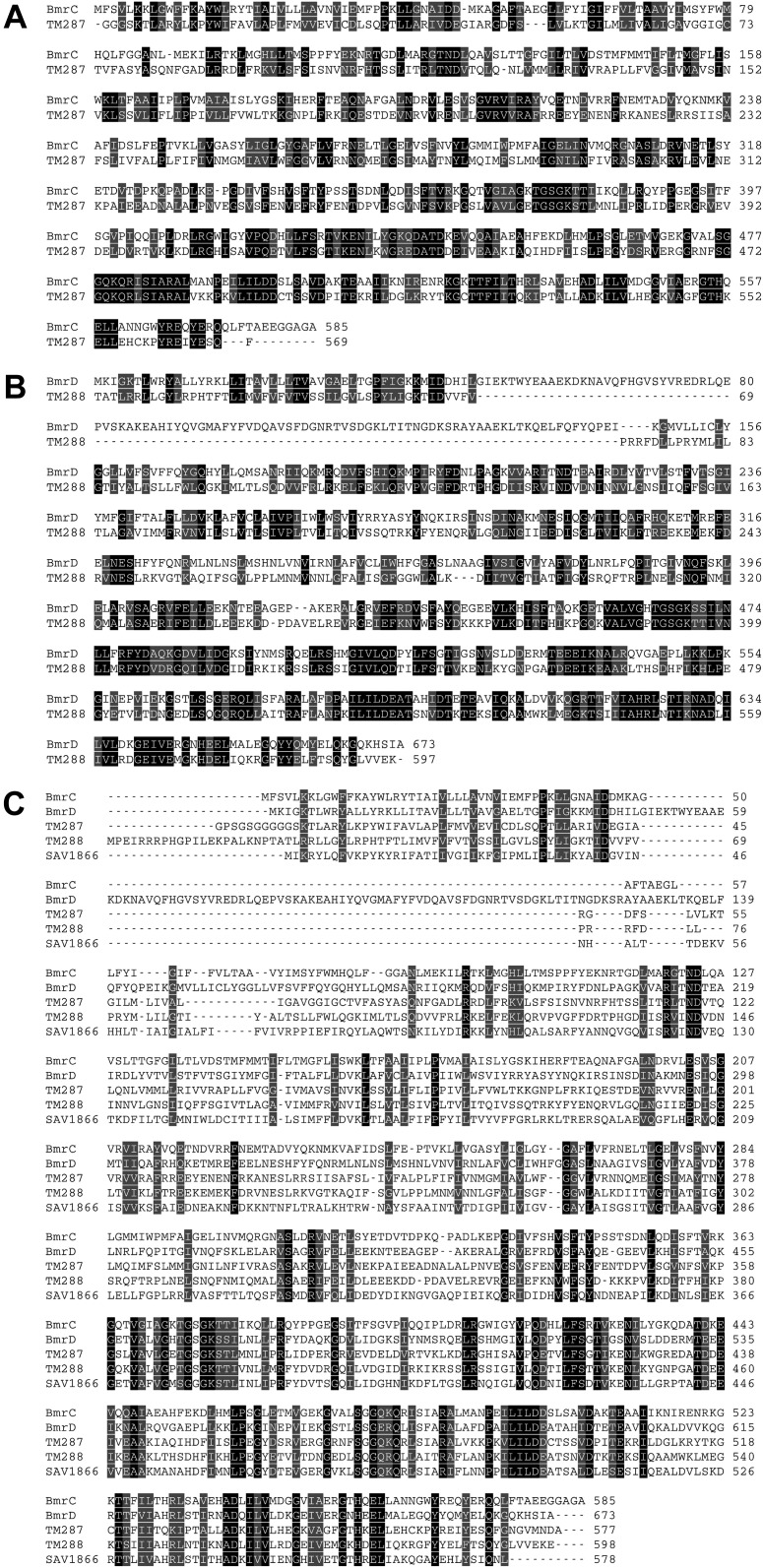
Sequence alignments. (**A**) Sequence alignment of BmrC with TM287. (**B**) Sequence alignment of BmrD with TM288. These sequence alignments were used as input for BmrCD homology modeling. (**C**) Multiple sequence alignment to identify the non-homologous region of BmrD for the homology model. The sequence of BmrC, BmrD, TM287, TM288 and Sav1866 were used to constrain the alignment of TM1 and an unconserved insertion in BmrD. The alignments were formatted using the Sequence Manipulation Suite (Stothard, 2000) web server. **DOI:** http://dx.doi.org/10.7554/eLife.02740.004

**Figure 1—figure supplement 2.**
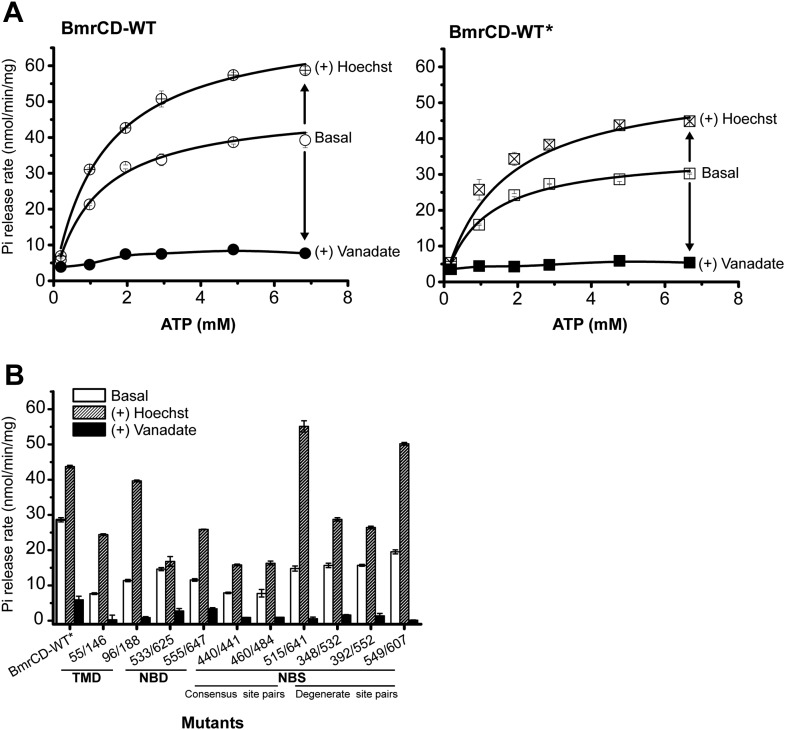
The ATPase activity of BmrCD-WT (wild type BmrCD), BmrCD-WT* (Cysteine-less BmrCD) and its spin-labeled mutants is stimulated by Hoechst33342 (hereafter referred as Hoechst) (10 µM) and inhibited by vanadate (1 mM). (**A**) Substitution of the native cysteines with alanines marginally reduces the *V*_max_ of ATP turnover. The solid line is a non-linear least-squares fit that yields *V*_max_ (48.52 ± 1.85 nmol/min/mg), *K*_m_ (1.19 ± 0.13 mM) for BmrCD-WT (basal); *V*_max_ (72.39 ± 2.86 nmol/min/mg), *K*_m_ (1.35 ± 0.14 mM) for BmrCD-WT (in presence of Hoechst) and *V*_max_ (36.02 ± 1.51 nmol/min/mg), *K*_m_ (1.08 ± 0.17 mM) for BmrCD-WT* (basal); *V*_max_ (56.86 ± 2.47 nmol/min/mg), *K*_m_ (1.6 ± 0.27 mM) for BmrCD-WT* (in presence of Hoechst 33,342). *V*_max_ and *K*_m_ values for BmrCD-WT, BmrCD-WT* and spin-labeled BmrCD are the average of three independent measurements. (**B**) All spin-labeled BmrCD pairs used for this study turnover ATP well above the background observed from inhibition by vanadate. *V*_max_ increased for all pairs following the addition of Hoechst although the level of stimulation was somewhat variable. **DOI:** http://dx.doi.org/10.7554/eLife.02740.005

**Figure 1—figure supplement 3.**
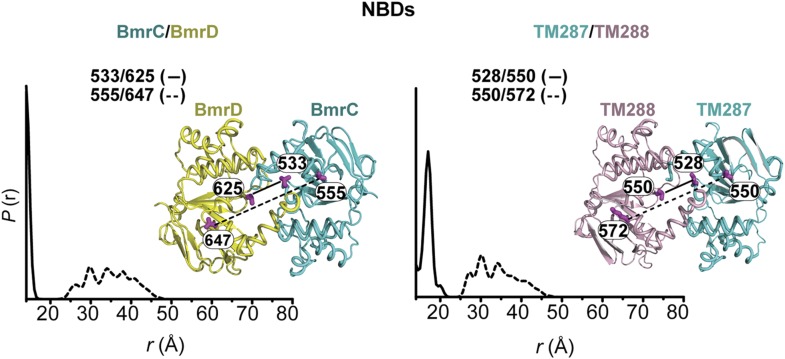
Predicted distance distributions between spin-labeled pairs monitoring the NBD interface. The distributions were calculated using the MMM package (Polyhach et al., 2011) for the BmrCD homology model and nucleotide-bound TM287/TM288 crystal structure. **DOI:** http://dx.doi.org/10.7554/eLife.02740.006

**Figure 1—figure supplement 4.**
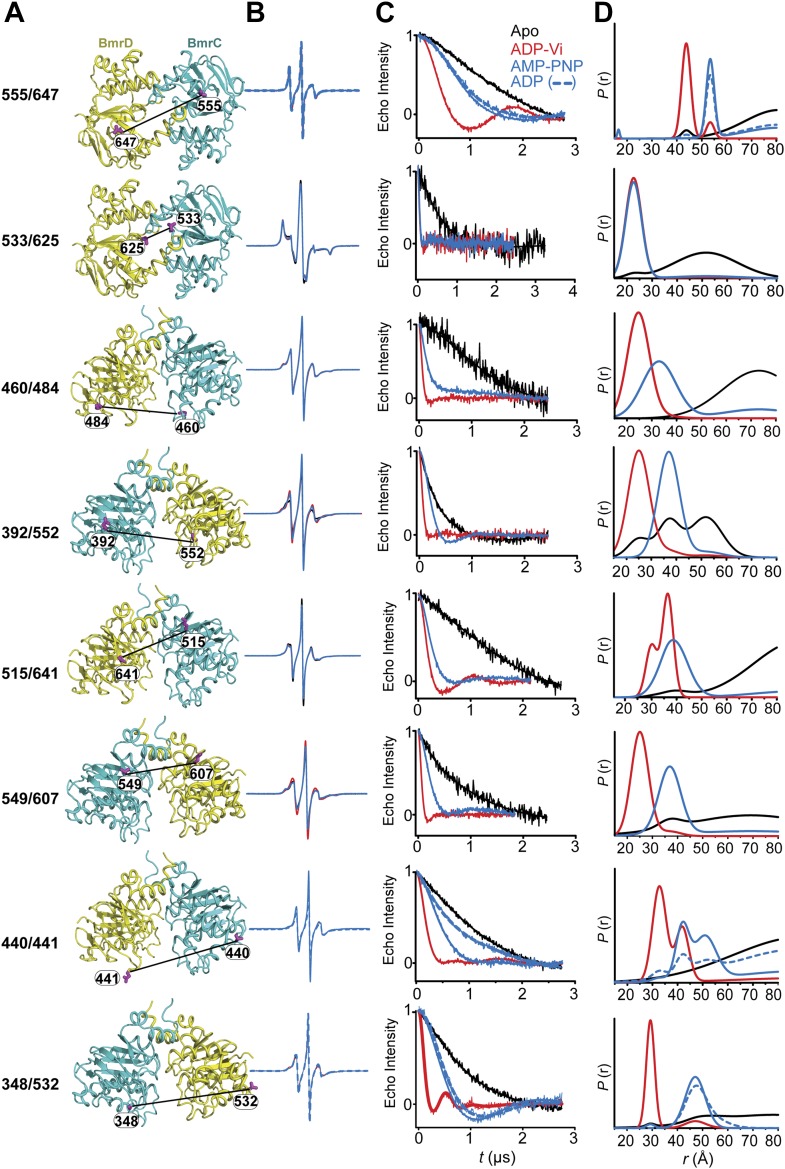
CW-EPR and DEER data analysis for spin-labeled pairs in the NBDs. (**A**) Close up view of BmrCD homology model highlighting the location of the spin-labeled pairs in the NBDs. (**B**) Superposition of the CW-EPR spectra demonstrates minimal changes in the lineshape, and by extension the spin label rotamer preferences. (**C**) Baseline-corrected DEER signals along with the fits corresponding to the distance distributions in panel (**D**). **DOI:** http://dx.doi.org/10.7554/eLife.02740.007

**Figure 1—figure supplement 5.**
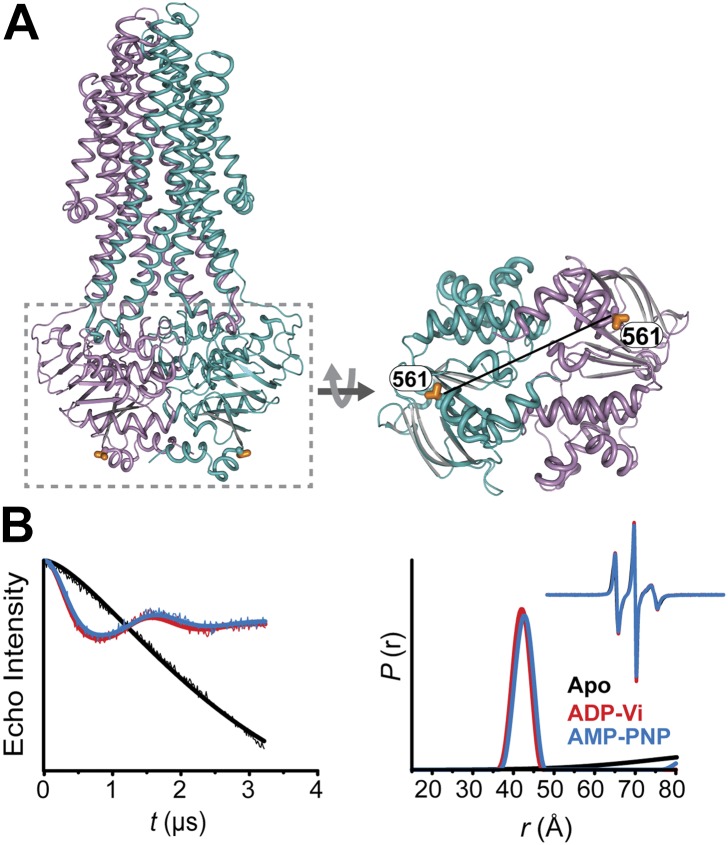
Conformational states of MsbA NBDs. (**A**) Ribbon representation of MsbA (PDB:3B60) showing the spin label pair 561 at symmetric sites across the NBD dimer interface (the transporter is turned 90° and NBD interface is viewed from cytoplasm). (**B**) DEER Echo (left panel) and corresponding distance distributions (right panel) revealing large amplitude movement upon trapping MsbA in the ADP-Vi state (red trace) and upon AMP-PNP binding (blue trace). Both conditions result in the same average distance demonstrating that ATP binding is sufficient for the formation of the NBD sandwich observed in the crystal structure. **DOI:** http://dx.doi.org/10.7554/eLife.02740.008

Distance distributions were determined by global analysis (Beechem, 1992) of the raw DEER data sets for each spin label pair by simultaneously fitting the distance components in the functional heterodimer and the background contribution from inter-dimer dipolar coupling as previously described (Brandon et al., 2012; Masureel et al., 2014) (‘Materials and methods’). This approach removes user bias in the baseline correction process which is critical for comparing populations under ATP turnover conditions. DEER distance distributions describe the distance probablilites between pairs of spin labels (Mchaourab et al., 2011). While the average distance can be related to the Cα-Cα distance between the two sites of spin label attachment (Islam et al., 2013), the distance changes deduced from shifts in the average distance can be interpreted as a direct measure of movement of the backbone (Mchaourab et al., 2011).

### Conformational states of the NBDs

In the absence of nucleotide or substrates, hereafter referred to as the apo state, BmrCD NBDs are disengaged as evident from distances between spin labels that are larger than those predicted on the basis of the nucleotide-bound TM287/288 crystal structure and the BmrCD homology model (Figure 1—figure supplement 3). Featureless DEER decays at spin label pairs 533/625 and 555/647, which were designed to monitor the NBD dimer interface, firmly reflect distances longer than 50 and 70 Å, respectively (black trace, Figure 1B,C, Figure 1—figure supplement 4). The average distance and broad distribution at 533/625 are similar to those observed at the equivalent MsbA site 539 (Borbat et al., 2007). Accordingly, they report a relatively large separation between the NBDs in the apo state similar to that of inward-open apo MsbA (Borbat et al., 2007; Zou et al., 2009) and are suggestive of considerable conformational flexibility.

ATP turnover followed by Vanadate (Vi) trapping stabilizes the transporter in an otherwise high-energy post-hydrolysis state (HES), also referred to as the transition state of ATP hydrolysis (Sharma and Davidson, 2000). The energy input from ATP hydrolysis leads to a large relative movement that brings the two NBDs closer together as manifested by the reduction in the average distances at 533/625 and 555/647 to about 25 and 45 Å, respectively (red trace, Figure 1B,C). The narrower distance distributions indicate a restriction of NBD conformational flexibility. Compared to the equivalent sites in MsbA (Borbat et al., 2007; Zou et al., 2009) (539, 561), these distance distributions are consistent with the formation of a closed NBD sandwich similar to that observed in the AMP-PNP bound crystal structures of MsbA (Ward et al., 2007) and Sav1866 (Dawson and Locher, 2007).

Binding of the non-hydrolyzable ATP analog AMP-PNP stabilizes a conformation distinct from that observed under trapped or apo conditions (blue trace, Figure 1C) and well outside the range predicted by the crystal structure and the homology model (Figure 1—figure supplement 3). This is in stark contrast to the ABC homodimer, MsbA, where ATP binding and HES trapping stabilize the same conformation of the NBD dimer (Figure 1—figure supplement 5). The 555/647 pair suggests an arrangement of the NBDs where the spin labels are separated by a longer average distance than in the HES but not as disengaged as apo (Figure 1C). These characteristics suggest that ATP binding, mimicked by AMP-PNP, nucleates the formation of an NBD dimer distinct from the canonical closed NBD sandwich observed in the crystal structures of nucleotide-bound MsbA (Ward et al., 2007) and Sav1866 (Dawson and Locher, 2007). Remarkably, a similar distance is observed in the presence of ADP (Figure 1C) suggesting that both nucleotides stabilize this intermediate state of the NBD dimer.

**Figure 2.**
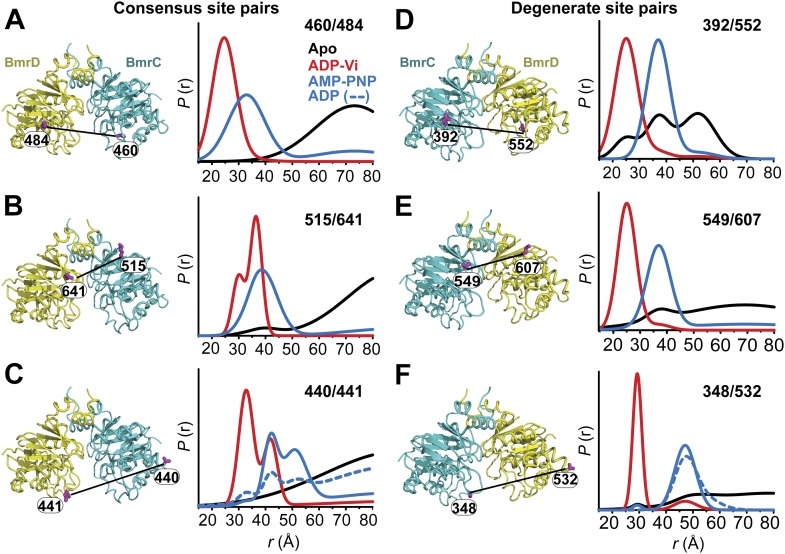
Structural asymmetry of the NBSs. (**A**–**C**) Close up side view of the NBD dimer and distance distributions for spin label pairs monitoring the consensus NBS. The overlapping distributions in the AMP-PNP bound and ADP-Vi trapped intermediates demonstrate that ATP binding can induce formation of the HES state. In contrast, distance distributions of structurally equivalent pairs monitoring the degenerate NBS (**D**–**F**) are predominantly non-overlapping. The views in (**D**–**F**) are obtained by rotating the views of **A**–**C** by 180°. See also Figure 1—figure supplement 2B and Figure1—figure supplement 4. **DOI:** http://dx.doi.org/10.7554/eLife.02740.009

ATP hydrolysis requires the assembly of the NBSs for optimal positioning of the catalytic residues of the conserved motifs (George and Jones, 2012). Therefore, we monitored the conformation of each NBS in the various catalytic intermediates of BmrCD outlined above. In order to measure distances across the NBSs, spin label pairs were introduced at non-symmetric sites in the BmrC and BmrD protomers. Three pairs of residues monitoring the degenerate and consensus NBSs (Figure 2) were selected to be structurally equivalent based on sequence alignment between BmrC and BmrD and verified by inspection of the structure of TM287/288 (Hohl et al., 2012) and the BmrCD homology model.

**Figure 2—figure supplement 1.**
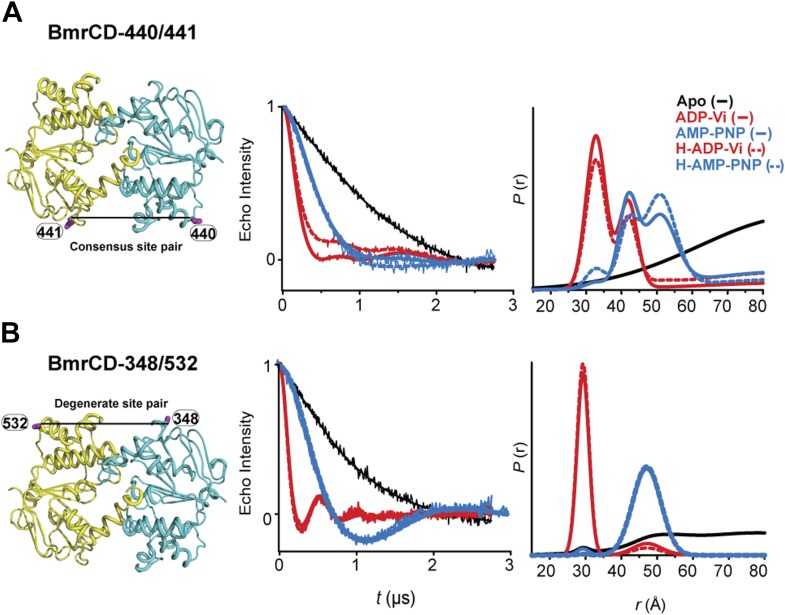
DEER data analysis for spin-labeled pairs in the NBSs. Close up view of BmrCD homology model highlighting the location of the pairs (**A**) (440/441), (**B**) (348/532) along with the baseline-corrected DEER decays and the corresponding distance distributions. Addition of Hoechst does not affect the distance distribution in detergent micelles. **DOI:** http://dx.doi.org/10.7554/eLife.02740.010

We found that nucleotide-induced changes in distance distributions reported at the NBSs (Figure 2) are consonant with those reported by the symmetry-related 555/647 which monitors NBD dimerization (Figure 1). Three distinct conformations for apo, AMP-PNP-bound and the HES intermediates are deduced from the shifts in distance distributions at the NBSs for all the pairs. However, the shape of the distributions uncovers fundamental structural differences between consensus and degenerate NBS. Consistently, spin labels reporting on the consensus NBS have overlapping distributions in the AMP-PNP-bound and trapped HES intermediates whereas at the degenerate site the distributions are by and large distinct (Figure 2, compare panels A, B, and C with D, E, and F). The breadth of the AMP-PNP distribution at the consensus NBS demonstrates that ATP binding enables a range of conformations to be populated that includes the HES. In contrast, ATP hydrolysis is required to observe an HES population at the degenerate NBS as inferred from the predominantly distinct distributions in the presence of AMP-PNP and ADP-Vi. In the presence of excess ADP, the degenerate NBS has a predominantly ATP-bound like conformation while the consensus NBS has a significant population of apo-like conformation (Figure 2, Figure 1—figure supplement 4). Both NBSs disengage in the absence of nucleotides consistent with the relative distance and conformational flexibility observed at the NBD interface (Figure 2, black traces). Addition of the transport substrate Hoechst (Torres et al., 2009) did not change the distance distributions of the AMP-PNP and ADP-Vi states (Figure 2—figure supplement 1).

### Transition of the TMDs from an inward- to an outward-facing conformation requires ATP hydrolysis

**Figure 3.**
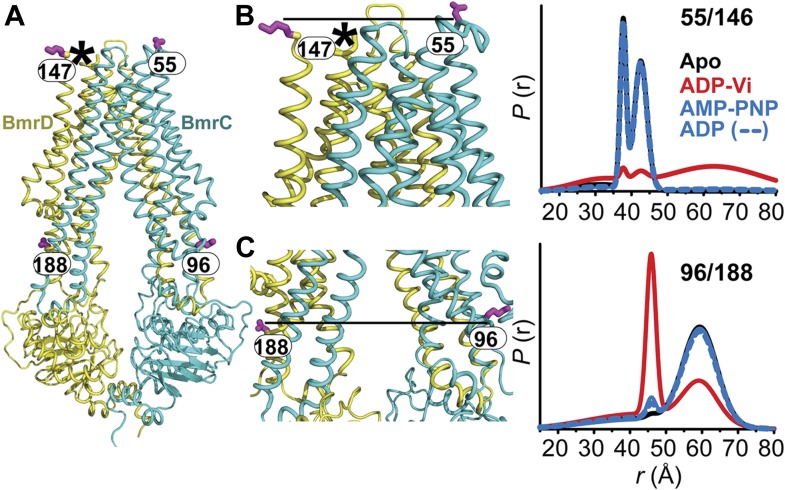
The inward- to outward-facing transition of the TMD requires ATP hydrolysis. (**A**) Ribbon representation of the BmrCD homology model showing the spin-labeled pairs designed to monitor the conformation of the TMD. Residue 147 is shown since 146 was not included in the homology model. ATP turnover and trapping with Vi, but not AMP-PNP binding, induces opening of (**B**) the extracellular side and (**C**) the closing of the intracellular side of the transporter. See also Figure 1—figure supplement 2B. **DOI:** http://dx.doi.org/10.7554/eLife.02740.011

Conformational changes in the TMDs, as a consequence of ATP binding and hydrolysis, were monitored by spin label pairs on the intracellular and extracellular sides of the transporter (Figure 3). These pairs were introduced at equivalent residues to MsbA spin label pairs which reported structural rearrangements associated with the inward- to outward-facing transition (Borbat et al., 2007). Specifically, the 55/146 pair monitors the extracellular loops between helices 1 and 2 at a structurally similar position to MsbA residue 61 while the pair 96/188 monitors the movement of the intracellular region of transmembrane helix 2 (TM2) similar to residue 103 in MsbA (Figure 3B,C).

**Figure 3—figure supplement 1.**
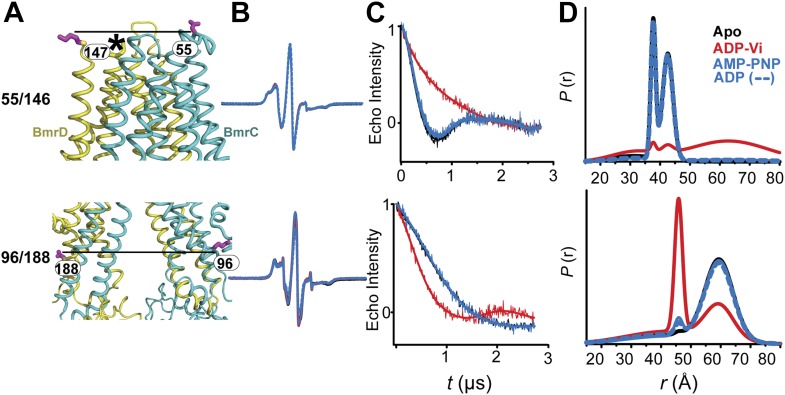
CW-EPR and DEER data analysis for spin-labeled pairs in the TMDs. (**A**) Close up view of BmrCD homology model highlighting the location of the spin-labeled pairs at the extracellular (upper panel) and intracellular (lower panel) side. * residue 146 was not modeled therefore residue 147 is displayed. (**B**) Superposition of the CW-EPR spectra demonstrates minimal changes in the EPR lineshape, and by extension the rotamer preferences of the spin label pairs, following addition of nucleotides. (**C**) Baseline-corrected DEER signals along with the fits corresponding to the distance distributions in panel (**D**). **DOI:** http://dx.doi.org/10.7554/eLife.02740.012

The pattern of average distances observed in the apo state of BmrCD is consistent with an open intracellular side and a relatively more closed extracellular side (Figure 3B,C, black trace, Figure 3—figure supplement 1). Furthermore, the intracellular side of apo BmrCD appears to be more closed than that of apo MsbA consistent with TM287/288 structure and the homology model. For instance, the distance between the symmetry-related residues 103 in the MsbA homodimer (previous work Borbat et al., 2007 and see below) is outside the range of measurable distances by DEER, at least 10 Å longer than the distance measured at the equivalent BmrCD pair, 96/188.

While ATP binding, mimicked by addition of AMP-PNP, induces the formation of a distinct NBD intermediate in BmrCD, we found that this conformational change is not propagated to the TMD. Spin label pairs at the intracellular and extracellular sides have identical distance distributions in apo and AMP-PNP-bound (Figure 3, black and blue traces respectively). This finding suggests relative flexibility between the NBD and TMD that allows large independent movement of the former upon ATP binding. In contrast, when ATP is hydrolyzed and ADP subsequently trapped by the addition of Vi, we observed concomitant shifts in the average distance at the intra- and extracellular sides of the transporter. Coupled to the formation of the NBD closed dimer, ATP hydrolysis induces closing of the intracellular side and opening of the extracellular side consistent with an alternating access mechanism involving inward-facing and outward-facing conformations. Compared to MsbA (residue 103) (Borbat et al., 2007) the distance change at the intracellular side is of smaller magnitude (approximately 15 Å for BmrCD 96/188 vs 25 Å for MsbA 103) primarily due to a closer distance between the TMDs in the apo conformation of BmrCD (Figure 3). More importantly, while conformational changes in MsbA are induced by the binding of AMP-PNP, the inward- to outward-facing transition in BmrCD requires ATP hydrolysis revealing a distinct power stroke between homodimers and heterodimers.

### Conformational dynamics in lipid bilayers

**Figure 4.**
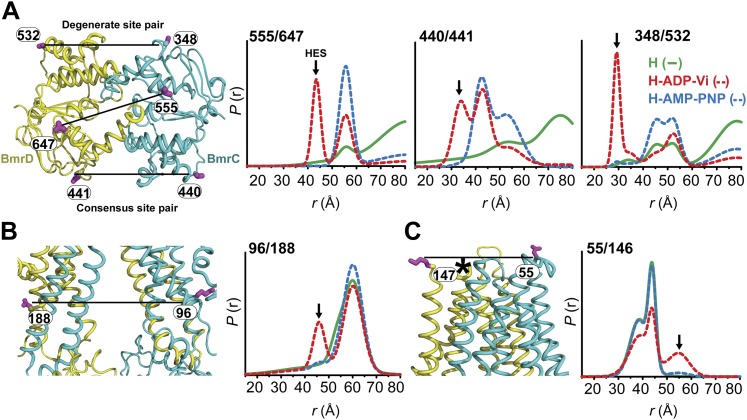
Conformational states of BmrCD in lipid bilayers. (**A**) Close up view from cytoplasmic side for spin label pairs monitoring the NBD dimer, the consensus NBS and the degenerate NBS along with the corresponding distance distributions. (**B**) Close up view from the membrane for spin label pairs monitoring the closing on intracellular side and (**C**) the opening on extracellular side of the transporter. The arrow points to the distance corresponding to HES. **DOI:** http://dx.doi.org/10.7554/eLife.02740.013

To test whether the substrate-coupled ATP turnover cycle in lipid bilayers involves the conformations identified in detergent micelles, we determined distance distributions in the NBD and TMD pairs following reconstitution of BmrCD double mutants in nanodiscs of PC/PA (Figure 4, Figure 4—figure supplement 1). This particular lipid mixture was selected on the basis of previous results showing optimal ATPase activity (Galián et al., 2011). Reconstitution in nanodiscs stimulated the ATP turnover rates of BmrCD-WT and BmrCD-WT* by 11–14-fold relative to detergent micelles (Figure 4, Figure 4—figure supplement 2). The rates were further stimulated two-fold in the presence of Hoechst.

**Figure 4—figure supplement 1.**
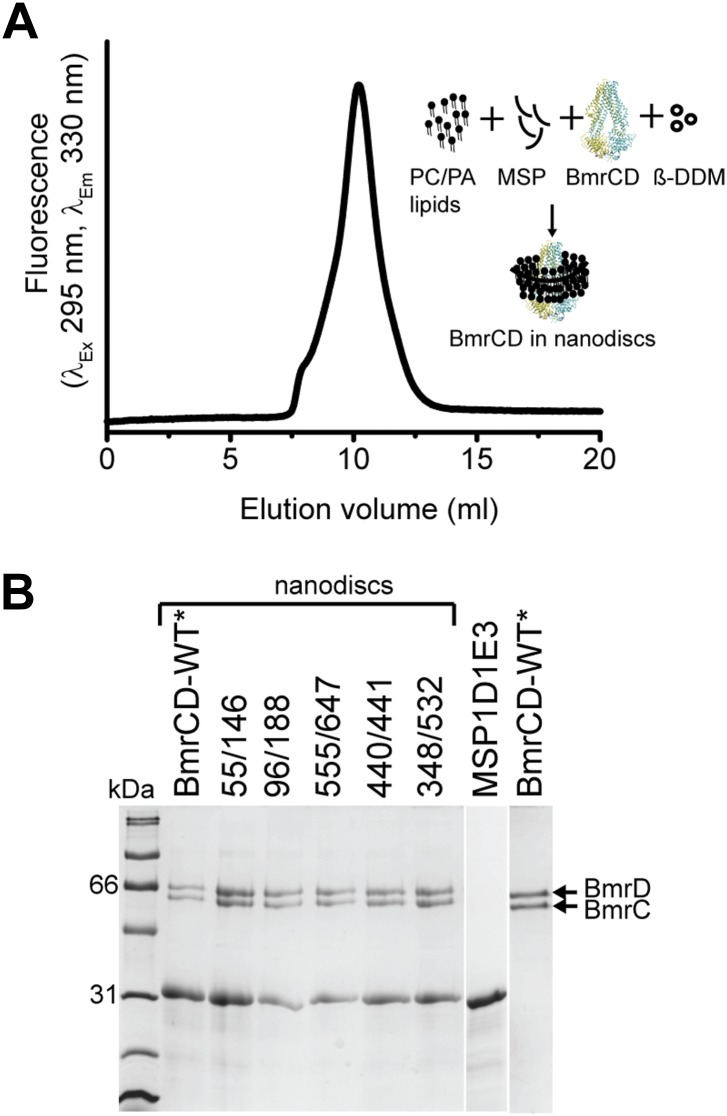
Reconstitution of BmrCD-WT* (Cysteine-less BmrCD) and its spin-labeled mutants in PC/PA nanodiscs. (**A**) Size-exclusion chromatography of BmrCD in nanodiscs and a cartoon depicting BmrCD-WT* assembly in nanodiscs. Similar size-exclusion chromatographs were obtained for spin-labeled mutants. (**B**) SDS-PAGE of BmrCD-WT* and its spin-labeled mutants assembled in nanodiscs showing the presence of MSP and BmrCD. **DOI:** http://dx.doi.org/10.7554/eLife.02740.014

**Figure 4—figure supplement 2.**
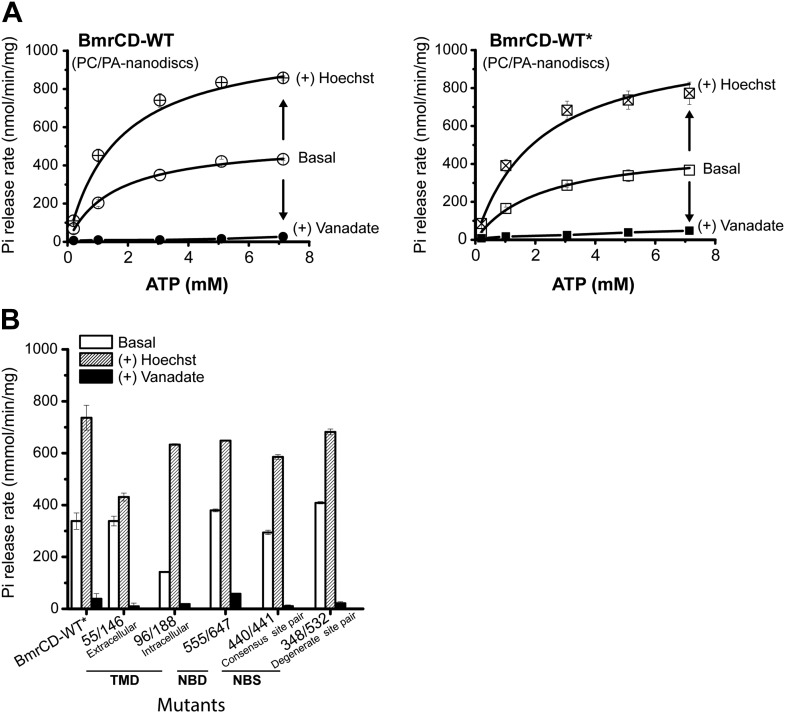
The ATPase activity of BmrCD-WT (wild type BmrCD), BmrCD-WT* (Cysteine-less BmrCD) and its spin-labeled mutants in PC/PA nanodiscs is stimulated by Hoechst (10 µM) and inhibited by vanadate (5 mM). (**A**) Reconstitution of BmrCD-WT, BmrCD-WT* and its spin-labeled mutants in PC/PA nanodiscs stimulates the basal ATPase activity. The solid line is a non-linear least-squares fit that yields *V*_max_ (527.6 ± 14.9 nmol/min/mg), *K*_m_ (1.53 ± 0.2 mM) for BmrCD-WT (basal); *V*_max_ (1065 ± 32.9 nmol/min/mg), *K*_m_ (1.65 ± 0.18 mM) for BmrCD-WT (in presence of Hoechst) and *V*_max_ (494.2 ± 45.41 nmol/min/mg), *K*_m_ (2.19 ± 0.39 mM) for BmrCD-WT* (basal); *V*_max_ (1067.2 ± 98.3 nmol/min/mg), *K*_m_ (2.13 ± 0.37 mM) for BmrCD-WT* (in presence of Hoechst). *V*_max_ and *K*_m_ values for BmrCD-WT, BmrCD-WT* and spin-labeled BmrCD are the average of three independent measurements. (**B**) All spin-labeled BmrCD pairs used for this study turnover ATP well above the background observed from inhibition by vanadate. *V*_max_ increased for all pairs following the addition of Hoechst although the level of stimulation was somewhat variable. **DOI:** http://dx.doi.org/10.7554/eLife.02740.015

**Figure 4—figure supplement 3.**
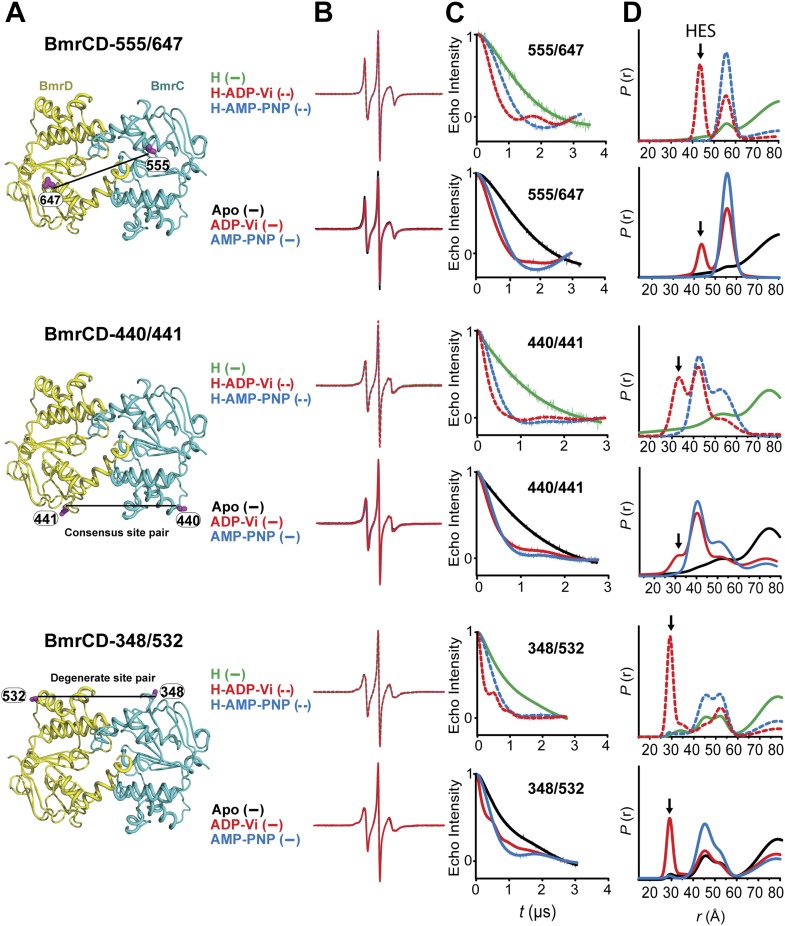
CW-EPR and DEER data analysis for BmrCD spin-labeled pairs reconstituted in nanodiscs. (**A**) Close up view of BmrCD homology model highlighting the location of the spin-labeled pairs in the NBDs and NBSs. (**B**) Superposition of the CW-EPR spectra demonstrates minimal changes in the lineshape, and by extension the spin label rotamer preferences. (**C**) Baseline-corrected DEER signals along with the fits corresponding to the distance distributions in panel (**D**). The arrow points to the distance corresponding to HES. **DOI:** http://dx.doi.org/10.7554/eLife.02740.016

**Figure 4—figure supplement 4.**
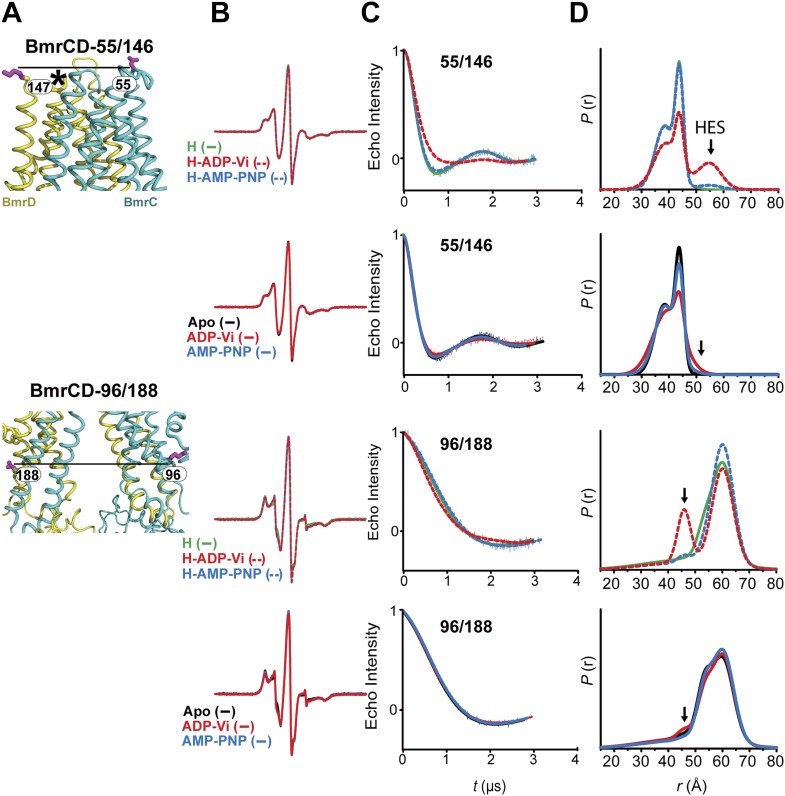
CW-EPR and DEER data analysis for BmrCD spin-labeled pairs reconstituted in nanodiscs. (**A**) Close up view of BmrCD homology model highlighting the location of the spin-labeled pairs at the extracellular (upper panel) and intracellular (lower panel) side. * residue 146 was not modeled therefore residue 147 is displayed. (**B**) Superposition of the CW-EPR spectra demonstrates minimal changes in the EPR lineshape, and by extension the rotamer preferences of the spin label pairs, following addition of nucleotides. (**C**) Baseline-corrected DEER signals along with the fits corresponding to the distance distributions in panel (**D**). The arrow points to the distance corresponding to the HES. **DOI:** http://dx.doi.org/10.7554/eLife.02740.017

Distance distributions in nanodiscs reveal components almost identical to those in detergent micelles (Compare Figure 4 with Figures 1–3,). Spin label pairs in the NBD show distinct distances in the apo state, AMP-PNP bound state, and the HES consistent with three conformations of the NBDs (Figure 4, top panel, Figure 4—figure supplement 3). In contrast, distance distributions of spin label pairs in the TMD identify only two conformational states corresponding to inward and outward-facing conformations (Figure 4, lower panel, Figure 4—figure supplement 4). ATP hydrolysis is required to transition between these states whereas BmrCD bound to AMP-PNP- is in the inward-facing conformation, in agreement with the observations in detergent micelles described above (Figure 3). Thus, not only is there a correspondence between conformational states in detergent micelles and lipid bilayers but also the power stroke is essentially identical. Remarkably, the transition between inward- and outward-facing conformations in nanodiscs shows a more stringent dependence on the presence of substrate (Figure 4, Figure 4—figure supplements 3 and 4). Thus, although ATP is turned over in the absence of substrate, transmission of this hydrolysis step is enhanced by the presence of the substrate Hoechst.

### Conformational dynamics under ATP turnover conditions reveal structural asymmetry at the NBSs

**Figure 5.**
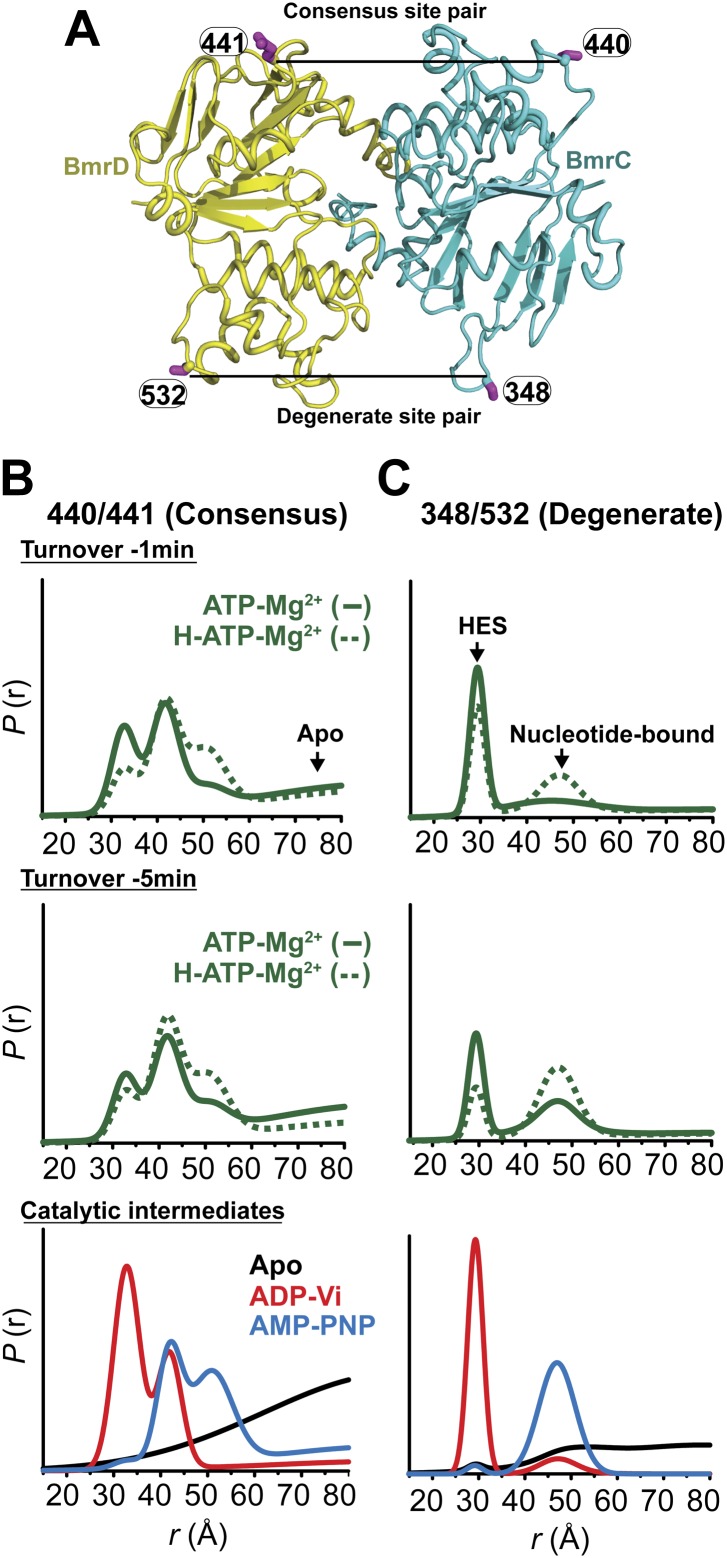
Structural asymmetry of the NBSs under turnover conditions. (**A**) Ribbon diagram of BmrCD NBDs showing the location of spin label pairs monitoring the consensus and the degenerate NBS (the NBD dimer is viewed from the cytoplasm, along the membrane normal). Following 1 or 5 min of incubation with 10 mM ATP at 30°C in (**B**) and (**C**) in presence (dashed traces) or absence (solid traces) of the substrate Hoechst (H), samples of spin-labeled BmrCD were immediately cooled to 4°C and then analyzed by DEER spectroscopy (Jeschke and Polyhach, 2007; Mchaourab et al., 2011). The two panels labeled ‘Catalytic intermediates’ are identical to those in Figure 2 and serve as a reference to assign the components in the distance distributions under turnover conditions. **DOI:** http://dx.doi.org/10.7554/eLife.02740.018

To frame the stable intermediates of the NBSs identified above in the context of the transport cycle, distance distributions were obtained under conditions that reflect cellular ATP concentrations and allow the transporter to sample intermediates associated with ATP turnover (Figure 5, Figure 5—figure supplement 1). Following incubation with ATP in the presence and absence of the substrate Hoechst, distance distributions were compared at two time points, where the ATP concentration is calculated to remain above the *K*_*m*_. At the degenerate NBS, we observed predominantly two-component distance distributions that reflect two distinct transporter populations (Figure 5C). By comparing distance distributions for the same spin label pair in trapped intermediates and under turnover conditions, the two components are identified as arising from nucleotide-bound (ATP or ADP) and HES-like states. The population ratio of the two components shifts from the HES-like towards the nucleotide-bound state with little if any apo population observed at the two time points. In contrast, multicomponent distributions at the consensus NBS (Figure 5B) show, in addition to the HES-like and nucleotide-bound states, clear evidence of an apo-like population where the two NBDs are locally disengaged.

**Figure 5—figure supplement 1.**
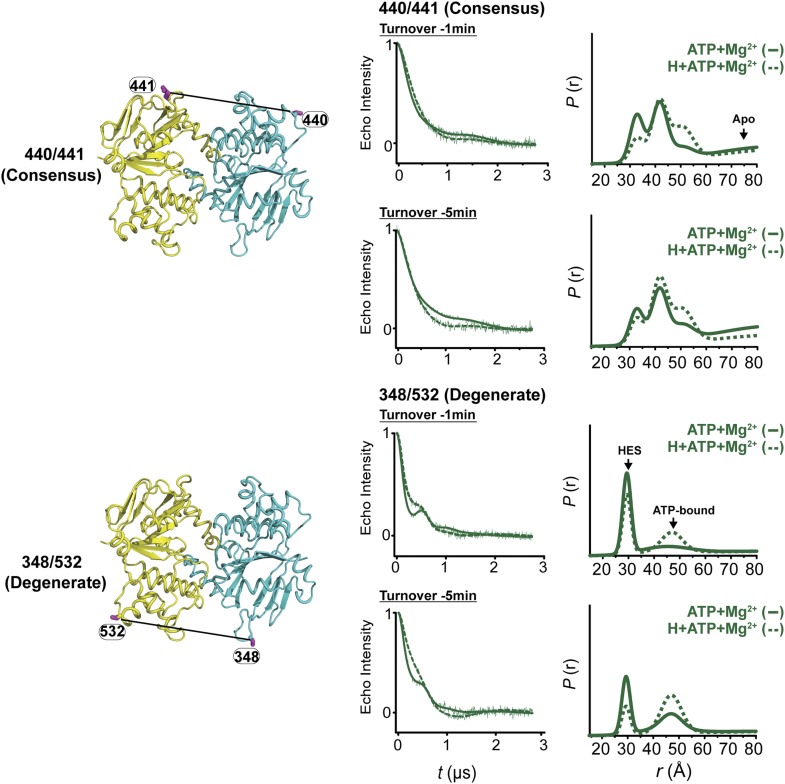
DEER data analysis for spin-labeled pairs in the NBSs under turnover conditions. Panels from left to right: location of spin-labeled pairs on BmrCD homology model, baseline-corrected DEER decays along with the fits and the resulting distance distributions. **DOI:** http://dx.doi.org/10.7554/eLife.02740.019

Thus, the two NBSs cycle between conformational states as ATP is bound, hydrolyzed and Pi and ADP are released. However, the differences in the population amplitude of these states reveal that the two NBSs do not have to concurrently be in the same catalytic intermediate. For example, while a significant population of the consensus NBS is in apo-like conformation, the degenerate NBS is predominantly in either the nucleotide-bound or HES-like conformations. Such an asymmetric disengagement of the signature and Walker A motifs at the consensus NBS, which requires their separation by more than 20–40 Å, is not structurally compatible with an HES-like conformation at the degenerate NBS. Therefore, the distributions under turnover conditions imply the existence of a transporter intermediate wherein the degenerate NBS is ATP-bound while the consensus NBS assumes an apo-like conformation. Similarly, we can infer the presence of another state of the transporter wherein the degenerate NBS is in a HES-like state and the consensus NBS is in the ATP-bound conformation.

Longer incubation times shift the distribution towards the ATP/ADP-bound population at the degenerate NBS (compare 1 and 5 min time points in Figure 5C) while at similar incubation times, the consensus site shows a considerably larger population in the ATP-bound and the apo states (Figure 5B). The addition of the substrate Hoechst, which accelerates the rate of ATP hydrolysis (Figure 1—figure supplement 2), simply shifts the populations at both NBSs towards those obtained at the longer incubation time. Thus, the substrate-coupled ATP turnover cycle of BmrCD involves the same conformational states sampled in the absence of substrate.

### Conformational dynamics under turnover conditions reveal a predominantly inward-facing conformation of the TMD

**Figure 6.**
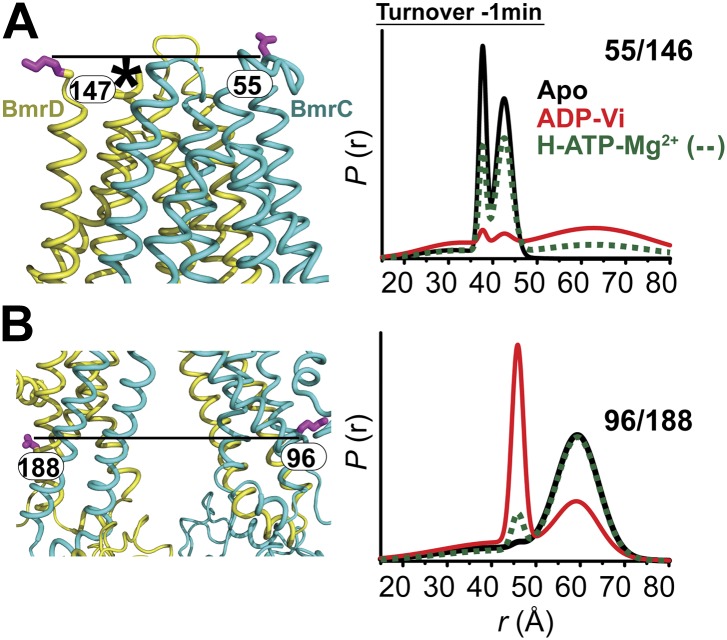
In the presence of excess ATP, the inward-facing state of BmrCD is favored. Distance distributions of spin label pairs monitoring the (**A**) extracellular and (**B**) intracellular side demonstrate that in the presence of 10 mM ATP the predominant population is inward-facing. **DOI:** http://dx.doi.org/10.7554/eLife.02740.020

Coupled to the ATP hydrolysis cycle of the NBSs, the TMD undergoes an inward- to outward-facing transition as evidenced by the time dependence of the distance distributions (Figure 6, Figure 6—figure supplement 1). Similar to the NBSs, the TMDs show multi-component distance distributions. However, the equilibrium favors the inward-facing conformation as demonstrated by the ratio of the two populations at high ATP concentrations. This presumably reflects the failure of ATP binding to switch TMD accessibility from inward-facing to outward-facing (Figure 3).

**Figure 6—figure supplement 1.**
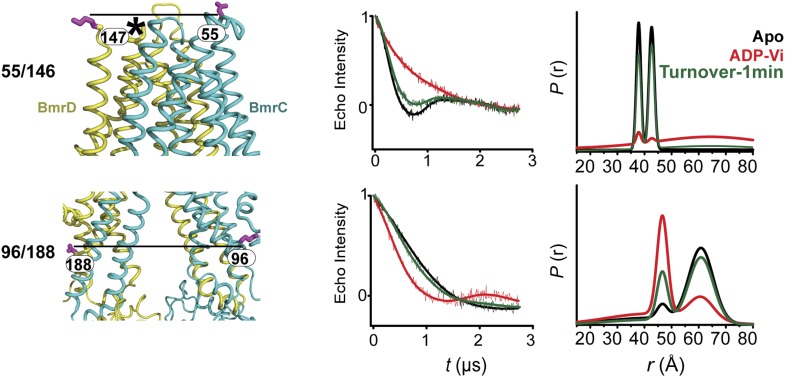
DEER data analysis for spin-labeled pairs in the TMDs under turnover conditions. Panels from left to right: location of spin-labeled pairs on BmrCD homology model, baseline-corrected DEER decays along with the fits and the resulting distance distributions. **DOI:** http://dx.doi.org/10.7554/eLife.02740.021

### MsbA inward- to outward-facing transition is driven by a two-state cycle of the NBDs

**Figure 7.**
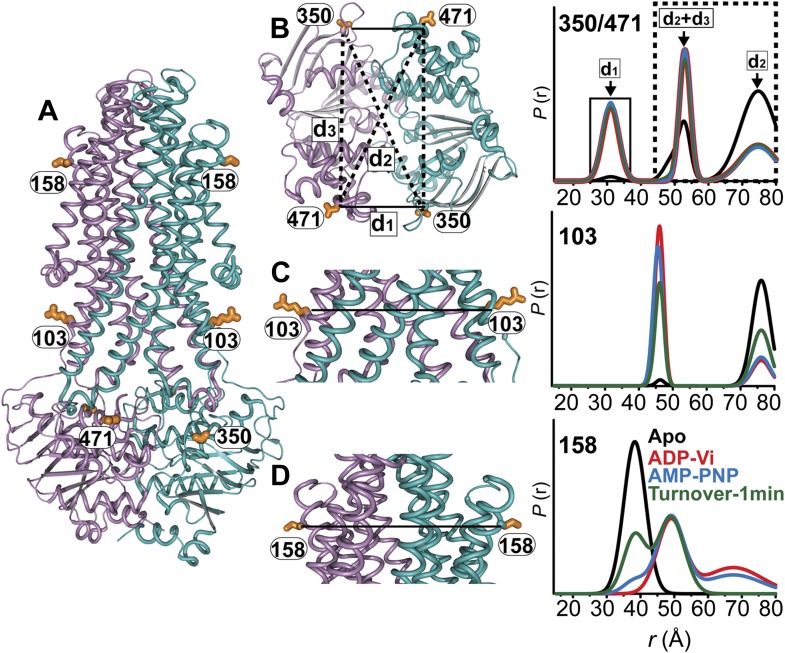
MsbA conformational dynamics under turnover conditions reveal a two-state equilibrium. (**A**) Ribbon representation of the AMP-PNP structure of MsbA (PDB:3B60) showing the spin-labeled pairs. (**B**) Binding of ATP induces the formation of the closed NBD dimer and concomitantly drives the transition to an outward-facing conformation of the TMD (**C** and **D**) In the presence of excess ATP, the equilibrium favors the outward-facing conformation (green trace). **DOI:** http://dx.doi.org/10.7554/eLife.02740.022

While the previous body of spin labeling data on MsbA establishes that ATP binding is the power stroke of transport (Dong et al., 2005; Zou and Mchaourab, 2009), we sought to conclusively investigate if the NBDs can adopt conformations besides the disengaged apo and the closed dimer stabilized by ATP binding or ADP-Vi trapping. Pairs of spin labels were introduced in each MsbA protomer with the purpose of monitoring the two NBSs following the design principles described above for BmrCD. Because MsbA is a homodimer, this strategy introduces four spin labels in the functional unit related by six distances (Figure 7A,B). If the NBD interface is twofold symmetric, three of these distances are unique (d1, d2, d3, in Figure 7B). Two distances relate labels at distinct sites: one is short range (d1) arising from pairs monitoring the NBS while the other represents symmetry-related sites that interact across the dimer interface and is considerably longer (d2). Therefore, in addition to considerations of ATPase activity (Figure 7—figure supplement 1) and surface localization, the two sites, 350 and 471, were selected to maximize the differences between spin labels reporting on the NBS conformation vs those reporting the distance between symmetry-related spin labels in the dimer. These selection criteria simplify the interpretation of the distance distributions and allow for the identification of the components that arise from dipolar coupling between spin labels across the NBS. However, the projection of the spin labels relative to the Cα breaks the symmetry leading to distinct distances in the 350/350 and 471/471 pairs. This complication does not affect the interpretation of the short component which monitors the NBSs.

**Figure 7—figure supplement 1.**
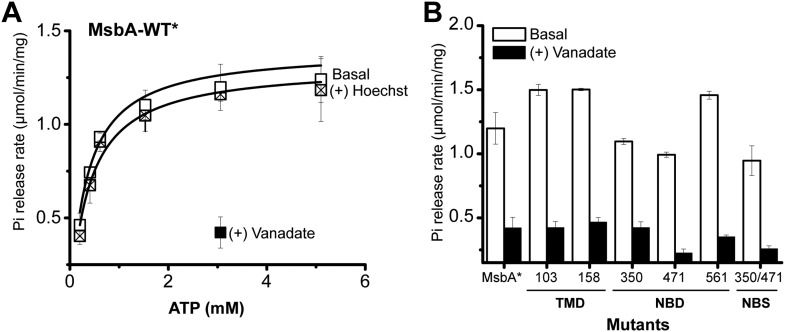
ATPase activity of MsbA. (**A**) Hoechst33342 (hereafter referred to as Hoechst) does not stimulate, but vanadate inhibits the ATPase activity of cysteine-less MsbA (MsbA-WT*) and (**B**) spin label mutants. ATPase activity of MsbA-WT* in the absence and presence of vanadate (1 mM) was measured as described in the methods section. The solid line is a non-linear least-squares fit that yields *V*_max_ (1.32 ± 0.059 µmol/min/mg), *K*_m_ (0.38 ± 0.06 mM) for MsbA-WT* (basal); *V*_max_ (1.4 ± 0.09 µmol/min/mg), *K*_m_ (0.33 ± 0.06 mM) for MsbA-WT* (in presence of Hoechst). *V*_max_ and *K*_m_ values for MsbA-WT* and spin label pairs are derived from three independent measurements. **DOI:** http://dx.doi.org/10.7554/eLife.02740.023

**Figure 7—figure supplement 2.**
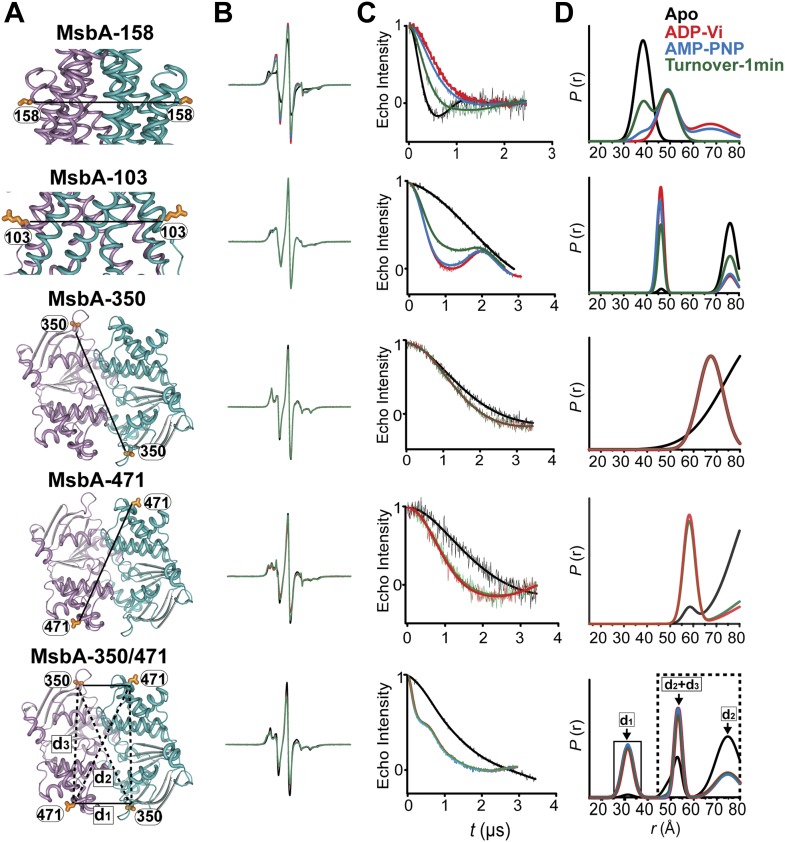
DEER data analysis for spin-labeled pairs. (**A**) Ribbon representation of the TMDs and NBDs of MsbA showing the spin label pairs (**B**) CW-EPR spectra (**C**) baseline-corrected DEER signals along with the fits and (**D**) the resulting distance distributions. **DOI:** http://dx.doi.org/10.7554/eLife.02740.024

Distance distributions were obtained in the apo, AMP-PNP-bound, ADP-Vi trapped and under turnover conditions (Figure 7B,C,D). As expected, MsbA NBDs completely dissociate in the absence of nucleotides as evidenced by the absence of a short distance component that would be expected from the assembly of the signature motifs into a NBS. In contrast, this short distance component is present to the same extent in the AMP-PNP-bound and ADP-Vi intermediates supporting the previous conclusion that ATP binding is the power stroke of transport for MsbA (Dong et al., 2005; Zou and Mchaourab, 2009; Figure 7B). We verified that this component arises from the formation of the NBS by measuring distance distributions for the corresponding single mutants (Figure 7—figure supplement 2). No other short distance component is detected as would be expected if the NBS were structurally asymmetric; although the detection of such asymmetry is limited by the intrinsic width of the distance distributions. Thus, we conclude that the two NBSs are structurally equivalent in the HES. The longer distance components in the distance distribution can be accounted for by the other distances (d2, d3) relating the two pairs of spin labels in the homodimer (Figure 7—figure supplement 2).

The two conformations of the NBDs, that is the disengaged and closed, are coupled to inward- and outward-facing conformations of the TMD respectively (Figure 7C,D). As previously reported, spin label pairs at the intra- and extracellular sides of the transporter report concurrent but opposite changes in average distance upon ATP binding suggesting that there is tight coupling between the movements of the NBDs and TMDs (Borbat et al., 2007; Zou et al., 2009) in contrast to the relative flexibility between the two domains in BmrCD. Under turnover conditions, the distance distributions were distinctly bimodal in the presence of ATP consistent with a partitioning of the transporter between inward- and outward-facing bound conformations. In contrast to BmrCD, the equilibrium of MsbA favors an outward-facing conformation. The predominance of the outward-facing conformation of the TMD is consistent with the persistence of the NBS short distance component under turnover conditions (Figure 7B).

## Discussion

The major novel finding of this paper is that sequence asymmetry in the NBDs shapes the mechanism of energy transduction in ABC exporters. Modifications of the Walker B, signature and/or switch motifs sequences, which lead to selective catalytic impairment of one of the NBSs in a subclass of ABC exporters (Gao et al., 2000; Aleksandrov et al., 2002; Zhang et al., 2006; Procko et al., 2009; Boncoeur et al., 2012), are associated with fundamental differences in the conformational dynamics of the NBDs and in the power stroke of transport. In contrast to the ATP-induced association/dissociation cycle of NBDs in bacterial homodimers reported previously (Borbat et al., 2007; Zou et al., 2009) and further confirmed here, the NBDs of heterodimers form structurally asymmetric dimers as a consequence of ATP binding and its subsequent hydrolysis. We uncovered conformations wherein the impaired NBS holds on to an ATP molecule while the consensus NBS disengages enabling product release and rebinding of ATP. Asymmetric NBD conformations were hypothesized from the elegant mechanistic analysis of Pgp by Senior et al. but were not directly detected (Tombline and Senior, 2005; Tombline et al., 2005). We propose that the distinct mechanism of heterodimers requires a less efficient coupling between the NBD and TMD, a ‘conformational leak’, to enable NBD movement without transmission to the TMDs. This would require a tuning of the sequences and interactions of the IH1 and IH2 coupling loops and possibly the cytoplasmic extension of TMs, regions hypothesized to form the transmission interface between the NBDs and TMDs of ABC transporters (Hollenstein et al., 2007; Oancea et al., 2009; Loo et al., 2013). There is a substantial impact of lipids on the coupling between the NBD and TMD. Transmission of the conformational changes to the TMD is more stringently dependent on the presence of substrate in lipid bilyaers. This finding is consistent with previous work revealing that lipids can shift the conformational preferences of ion-coupled transporters (Hanelt et al., 2013).

This novel insight was derived from experiments that monitor transporter conformational states under turnover conditions. Unlike previous studies, which investigated exclusively stable intermediates (Borbat et al., 2007; Zou et al., 2009), here the mechanistic implications of these intermediates were challenged under substrate and nucleotide conditions that mimic those of the cell. Another unique experimental design is the selective monitoring of individual NBSs rather than the NBD interface facilitated by the heterodimeric nature of BmrCD. NBS distance distributions capture the interconversion of the transporter between conformations corresponding to those observed under trapped conditions, thereby establishing the direct relevance of the trapped conformations to the substrate-coupled ATPase cycle. Furthermore, the observation of a steady state population of apo-like conformation of the consensus NBS, even in the presence of excess ATP, prompts us to conclude that disengagement of this NBS is required for ADP-Pi dissociation and subsequent binding of ATP, thereby establishing the mechanistic significance of a local apo-like conformation.

**Figure 8.**
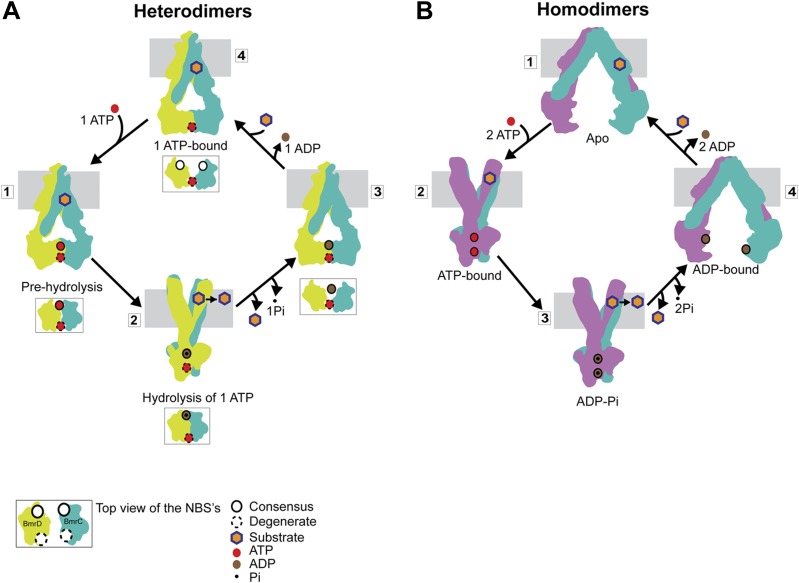
Distinct mechanisms of ABC heterodimers and homodimers. (**A**) Asymmetric hydrolysis of ATP in an ABC heterodimer. The conformation of the transporter and a top view of the NBD dimer are shown for each catalytic state. Substrate binding can precede or follow ATP binding to the NBSs. The cycle is triggered when ATP is bound at both NBSs (1). The top view of the NBDs shows the partially engaged ATP-bound intermediate state. Hydrolysis proceeds preferentially at the consensus NBS (2) driving substrate extrusion and leading to another asymmetric state of the NBD wherein the degenerate NBS is ATP-bound while the consensus NBS is ADP-bound (3). Dissociation of ADP occurs via a local apo-like conformation of the consensus NBS (4) thereby enabling a new transport cycle. (**B**) Two-state cycle of an ABC homodimer. Here substrate must precede ATP binding, which induces the parallel formation of the closed NBD and the outward-facing state of the TMD. Sequence symmetric NBSs hydrolyze two ATP molecules prior to disengagement of the NBDs. **DOI:** http://dx.doi.org/10.7554/eLife.02740.025

Our results can be framed in a model of how asymmetric ATP binding and hydrolysis in the NBDs drive transition of the TMDs between inward- and outward-facing conformations (Figure 8A). Distinguishing features of this model include the formation of structurally asymmetric conformations of the NBDs and the association of the transporter power stroke with ATP hydrolysis. In the presence of cellular ATP concentrations, BmrCD will have at least one ATP molecule bound, most likely at the degenerate NBS as evidenced by the fact that an apo-like conformation was nearly undetectable at this NBS in the presence of excess ATP. Such an ATP-bound resting state does not hinder substrate access to the putative binding chamber at the TMD interface, since ATP binding does not induce closure of the cytoplasmic side of BmrCD. ATP binding to the consensus NBS yields an intermediate with two ATP molecules bound (‘1’ in Figure 8A). We refer to this conformation of the NBD dimer (Figure 2) as a pre-hydrolysis intermediate reminiscent of the pre-hydrolysis complex observed in a crystal structure of the maltose transporter, MalK (Oldham and Chen, 2011). In this conformation, the NBDs are engaged but the NBSs have not formed the canonical configuration and are therefore not catalytically competent. ATP turnover is initiated by fluctuations at the consensus NBS which enables transition to a hydrolysis-competent configuration (HES-like) as deduced from the overlapping distance distributions in Figure 2. This is a relatively minor population of the transporter which likely explains the relatively slow ATP turnover by BmrCD (Figure 1—figure supplement 2). ATP hydrolysis switches TMD accessibility thereby catalyzing the extrusion of the substrate. Turnover rates are stimulated if the substrate binds prior to hydrolysis but there is a considerable basal consumption of ATP (Figure 1—figure supplement 2). While there is evidence of ATP turnover by impaired NBSs, consideration of the reaction mechanism as well as previous biochemical studies of ABC heterodimers support an intrinsically slower rate at the degenerate NBS (Procko et al., 2006; Zutz et al., 2011; Boncoeur et al., 2012). Therefore, ADP and Pi are released from the consensus site prior to hydrolysis at the degenerate site enabling multiple rounds of asymmetric ATP hydrolysis without the complete disengagement of the NBDs to an apo conformation.

Slow ATP hydrolysis at the degenerate NBS would require release of ADP and Pi and rebinding of ATP most likely through the population of a local apo-like conformation. Whether ATP hydrolysis at the degenerate NBS could occur prior or concomitant with release of ADP and Pi from the consensus NBS cannot be conclusively addressed from our data. However, the negligible population of apo-like degenerate NBS and the fact that ADP-bound NBDs form a partially engaged dimer argue against significant population of an apo state of the transporter where the two NBDs are separated by more than 50 Å. Thus, this ‘global’ apo state (as in Figure 1) appears not to be mechanistically relevant in the ATP hydrolysis cycle of ABC heterodimers. We propose that ADP-Pi release, as a consequence of infrequent hydrolysis at the degenerate site, occurs while ATP is bound at the consensus NBS.

If the degenerate site is not turning over ATP at levels comparable to the consensus NBS, how is the significant HES-like population observed under turnover conditions (Figure 5B) accounted for? We speculate that hydrolysis at the consensus NBS requires transition of the degenerate NBS to an ATP-occluded state along the lines of the model proposed for Pgp on the basis of experiments with catalytically impaired NBSs (Tombline and Senior, 2005). This state would relax to the nucleotide-bound conformation following hydrolysis at the consensus NBS (state 3 in Figure 8A).

The mechanistic elements of ATP turnover for ABC heterodimers and homodimers are contrasted in Figure 8. For the latter, the NBSs are identical in sequence and there is no experimental evidence of catalytic inequivalence. Analysis of MsbA conformational dynamics reported previously (Dong et al., 2005; Borbat et al., 2007; Zou and Mchaourab, 2009) and extended here is entirely consistent with a model of turnover that invokes engaged and disengaged NBD states, symmetric NBS conformations, and tight structural coupling between the NBDs and the TMDs. We conclude from the comparative analysis of BmrCD and MsbA conformational cycles that the controversy regarding NBD disengagement vs constant contact (George and Jones, 2012) is a consequence of extrapolation of models between mechanistically distinct classes of ABC exporters.

The structural mechanism of ABC heterodimers described here is a remarkable example of variations on a common conformational cycle in the context of conserved transporter architecture. While all ABC transporters derive energy from an ATP binding and hydrolysis cycle, fundamental differences exist in the regulation of ATP turnover by catalytic elements and/or structural intermediates as evidenced here. Although it was anticipated that the inward- to outward-facing transition of ABC exporters would follow the structural mechanics defined by the structures of MsbA (Ward et al., 2007), we show in this work that coupling of the TMD transition to the ATPase cycle is not identical between subclasses of exporters. Evidence of more variation in the coupling mechanism has emerged recently from a structure of a human ABC homodimer where the NBSs are disengaged even in the presence of AMP-PNP (Shintre et al., 2013). Finally, if indeed TMD conformational changes require hydrolysis of a single ATP molecule in heterodimers, it raises the conundrum of why two molecules are hydrolyzed in ABC homodimers to achieve what would appear to be similar type of mechanical work. Addressing these questions is the next frontier in understanding the mechanism of ABC exporters.

## Materials and methods

### Homology modeling of BmrCD

A homology model of the inward-facing BmrCD was constructed with MODELLER (Marti-Renom et al., 2000), using the crystal structure of the asymmetric ABC transporter TM287/288 (PDB: 3QF4) (Hohl et al., 2012) as the template. In order to correctly map the consensus and degenerate nucleotide-binding sites, TMD287 was chosen as the template for BmrC and TMD288 as a template for BmrD. The alignment between BmrC and TM287 was directly generated by pairwise Blastp (Figure 1—figure supplement 1A). However, due to low sequence identity, the homology of the first ∼150 amino acids of BmrD could not be determined through direct pairwise alignment. The BmrD/TM288 alignment (Figure 1—figure supplement 1B) was instead obtained by manually optimizing a multiple alignment involving sequences of BmrC, BmrD, TM287, TM288, and SAV1866 (Dawson and Locher, 2006; Figure 1—figure supplement 1C), which was generated using Clustal-Ω (Sievers et al., 2011). In the multiple alignment, the N-terminal region of BmrD (Met1-Leu48) is aligned to the elbow helix and the TM1 of other ABC transporters, followed by a heterologous insertion of ∼100 residues. Assuming the aligned sequence within a transmembrane helix is ungapped, the BmrD alignment near the heterologous insertion was then manually optimized by ungapped extension from the aligned sequence immediately C-terminal to the inserted region, which covers most of the TM2 of TM288. Similarly, the alignment of BmrD/TM288 near the TM5 (Asn333-Gly365 of BmrD) was also manually optimized due to its lower local sequence identity and the presence of inserted residues in the transmembrane region of the BmrD sequence. The inserted region of BmrD (Cys49–Ile146) was not modeled due to the lack of homologous sequence in any protein structure. The final sequence alignment used for homology modeling is shown in (Figure 1—figure supplement 1B). To maintain the structural stability and the global conformation during the refinement of the initial homology model, Cα atoms in ungapped aligned regions (Tyr15-Asp46, Leu57-Gly84, Leu93-Asp124, Leu137-Leu316, Gly335-Gln573 of BmrC; and Met1-Ile47, Phe162-Arg328, Ser368-Glu411, Arg429-Ala673 of BmrD) were excluded from the refinement, that is, their positions remain fixed in the final model.

### Cloning, expression, purification and labeling of BmrCD

Cysteine-less BmrCD (BmrCD-WT*) was constructed from wild type BmrCD (BmrCD-WT) in pET21b(+) (a kind gift of Dr JM Jault) by substitution of three native cysteines of BmrD by alanines using QuikChange site directed mutagenesis (Stratagene, La Jolla, USA). BmrCD-WT* template was then used to make double cysteine mutants. All substitutions were confirmed by DNA sequencing. BmrCD-WT, BmrCD-WT* and cysteine mutant plasmids were transformed into *E. coli* BL21(DE3) cells. A single transformant colony was inoculated into 20 ml LB for the primary culture which subsequently was used to start the main culture in 1 l minimal media supplemented with glycerol (0.5%), thiamin (2.5 μg/ml), ampicillin (100 μg/ml), MgSO_4_ (1 mM), and 50 × MEM amino acids (1 ml). Cultures were grown at 37°C with shaking to an OD_600_ of 1.2, and then expression of BmrCD was induced by addition of 0.7 mM isopropyl β-D-1-thiogalactopyranoside. BmrCD cultures were incubated at 25°C with shaking for another 5.5 hr. The cells were harvested by centrifugation and stored at −80°C. *E. coli* cell pellets were resuspended in 20 ml of lysis buffer (50 mM Tris–HCl, 5 mM MgCl_2_, pH 8.0), including 10 mM DTT, 10 μg/ml DNAase, 0.1 mM PMSF, 1/3 of a Complete EDTA-free protease inhibitor cocktail tablet (Roche, Indianapolis, USA) and were lysed by five passes through an Avestin C3 homogenizer at 15-20,000 PSI. The lysate was centrifuged at 9000×*g* for 10 min to remove cell debris and the membranes were isolated by ultracentrifugation at 200,000×*g* for 1 hr. Membranes were solubilized in resuspension buffer (50 mM Tris–HCl, 100 mM NaCl, 15% [vol/vol] glycerol, pH 8.0) including 1 mM DTT, 1% wt/vol n-dodecyl-β-D-maltopyranoside (β-DDM) with constant stirring on ice for 1 hr. Solubilized membranes were then centrifuged at 200,000×*g* for 45 min to 1 hr to remove insoluble particulates. The solubilized fraction was then incubated for 1 hr with 300 µl of pre-washed Ni-NTA resin (QIAGEN, Venlo, Limburg) pre-equilibrated with Ni buffer (50 mM Tris–HCl, 100 mM NaCl, 15% [vol/vol] glycerol, 0.05% β-DDM, pH 8.0). BmrCD bound Ni-NTA resin was loaded onto a column, washed with five column volumes of Ni buffer containing 20 mM imidazole and was eluted with 250 mM imidazole. Eluted BmrCD was incubated with a 20-fold excess of (1-Oxyl-2,2,5,5-tetramethylpyrroline-3-methyl) methanethiosulfonate (MTSSL, Enzo Life Sciences, Farmingdale, USA) for 4 hr at 23°C and placed on ice for ∼12 hr. The labeled protein was then separated from free label by size-exclusion chromatography on a Superdex 200 column in buffer containing 50 mM Tris–HCl, 150 mM NaCl, 10% (vol/vol) glycerol, 0.02% β-DDM, pH 7.4. BmrCD concentration was determined by absorbance at 280 nm (Mean extinction coefficient = 68,077.5 M^−1^ cm^−1^).

### Cloning, expression, purification and labeling of MsbA

Cysteine-less MsbA (MsbA-WT*) template (Dong et al., 2005) was used to generate single and double-cysteine substitutions. MsbA-WT* and cysteine substituted mutants were expressed, purified and labeled as previously described (Smriti et al., 2009; Zou and Mchaourab, 2009).

### Preparation of membrane scaffold protein

Membrane scaffold protein (MSP1D1E3) is expressed and purified as described earlier (Boldog et al., 2007) with the following modifications. Briefly, MSP1D1E3 gene in pET-28a(+) was obtained from Genscript (Piscataway, USA) and transformed in *E. coli* BL21(DE3) cells. A dense starter culture was used to inoculate secondary culture of 500 ml Terrific broth supplemented with 30 μg/ml of kanamycin. Cultures were grown at 37°C with shaking to an OD_600_ of ∼2.2–2.5, and then expression of MSP1D1E3 was induced by addition of 1 mM IPTG. Cultures were further grown for 4 hr at 37°C, and cells were harvested by centrifugation. Cell pellets were resuspended in 15 ml of lysis buffer (20 mM sodium phosphate, 1% Triton X-100, pH 7.4), including 1 mM PMSF, 1/3 of a Complete EDTA-free protease inhibitor cocktail tablet (Roche) and were lysed by sonication. The lysate was centrifuged at 30,000×*g* for 30 min, and the supernatant was loaded onto a Ni-NTA column equilibrated with lysis buffer, followed by washing with four bed volumes of wash buffer-1 (40 mM Tris/HCl, 0.3 M NaCl, 1% Triton X-100, pH 8.0), four bed volumes of wash buffer-2 (wash buffer-1 with 50 mM sodium cholate), four bed volumes of buffer A (40 mM Tris/HCl, 0.3 M NaCl, pH 8.0), four bed volumes of buffer A containing 20 mM imidazole, and eluted with buffer A containing 300 mM imidazole. The eluted MSP1D1E3 was passed over a desalting column into MSP buffer (50 mM Tris, 0.1M NaCl, 0.5 mM EDTA, pH 7.5) and the concentration was determined by absorbance at 280 nm (extinction coefficient = 29,910 M^−1^ cm^−1^).

### Preparation of lipid mixture for nanodiscs

PC (L-α phosphatidylcholine) and PA (L-α phosphatidic acid) (Avanti Polar Lipids, Alabaster, USA) were combined in a 9:1 molar ratio, dissolved in chloroform, evaporated to dryness on a rotary evaporator and desiccated overnight under vacuum. The lipids were hydrated in MSP buffer containing 0.5% (wt/vol) ß-DDM, filtered through 0.2 µm polycarbonate membrane (Whatman, Florham Park, USA) and stored in small aliquots at −80°C.

### Reconstitution of BmrCD in nanodiscs

For reconstitution into nanodiscs, purified BmrCD* or spin-labeled proteins in ß-DDM micelles were mixed with PC/PA lipid mixture, MSP1D1E3 and ß-DDM in the following molar ratios: lipid:MSP1D1E3, 120:1; MSP1D1E3:BmrCD, 3:1; ß-DDM:lipid, 5:1. Mixtures were rocked at room temperature for 30 min. Biobeads (0.8–1 g/ml) were then added to the solution and incubated overnight at 4°C with rocking. The nanodiscs assembly solution was filtered using 0.45 µm filter to remove biobeads. Full nanodiscs were separated from empty nanodiscs by size-exclusion chromatography. Nanodiscs were concentrated using Amicon Ultra-50K centrifugal filter units (Millipore, Billerica, USA). Nanodiscs having BmrCD* or spin-labeled mutants were then characterized using SDS-PAGE to verify reconstitution and estimate reconstitution efficiency. In another measure, concentration of spin-labeled mutants in nanodiscs was determined as described previously (Zou and McHaourab, 2010) by comparing the intensity of the integrated CW-EPR spectrum to that of the same mutant in detergent micelles.

### ATPase assay

The specific ATPase activity of BmrCD and MsbA was determined as previously described (Smriti et al., 2009) with the following modifications. Briefly, BmrCD in detergent micelles (20 µg), in nanodiscs (1 µg) and MsbA in detergent micelles (1 µg) samples were incubated with increasing concentrations of ATP at 30°C (detergent micelles), 37°C (nanodiscs) for 30 min and at 37°C for 20 min respectively in presence or absence of Hoechst and vanadate. The reaction was stopped by adding 1% SDS and the color was developed using a 1:1 solution of ammonium molybdate (2% in 1M HCl) and ascorbic acid (12% in 1M HCl). The absorbance of samples was measured at a wavelength of 850 nm on a BioTek Synergy H4 microplate reader. The amount of phosphate released was determined by comparison to inorganic phosphate standards.

### CW-EPR and DEER spectroscopy

For CW-EPR, spin-labeled BmrCD and MsbA samples were loaded in capillaries and spectra were collected on a Bruker EMX spectrometer using 10 mW microwave power level and a modulation amplitude of 1.6 G. DEER spectroscopy was performed on a Bruker 580 pulsed EPR spectrometer operating at Q-band frequency (33.9 GHz) with a standard four-pulse protocol at 83 K (Jeschke, 2002). A 30% (wt/wt) glycerol was added to samples as a cryoprotectant.

Raw DEER decays were analyzed using home-written software operating in the Matlab environment. The software implements a number of well-established approaches to DEER data analysis (Sen et al., 2007; Brandon et al., 2012). The motivation for developing this software was to carry out global analysis (Beechem, 1992) of the DEER decays obtained under different conditions for the same spin label pair. The distance distribution is assumed to consist of a sum of Gaussians, the number of which is determined based on a statistical criterion. The optimal number of Gaussian components and the center, width and amplitude of each component were allowed to vary between conditions. The slope of the background was assumed to be uniform across the data set but was fit rather than set by the user. Care was taken to ensure that the concentration of samples for the same spin label pair were identical within experimental error. The statistical significance between fits with increasing number of Gaussian distributions was determined using an F test. The root-mean squared difference between the data and fit was minimized using the trust-region-reflective algorithm implemented in the MATLAB routine ‘lsqnonlin’. We compared results from this analysis to those obtained by using the package DeerAnalysis 2011 (Jeschke et al., 2006) for data obtained in the trapped conditions (Figures 1–3) and found that the resulting distributions are very similar. The main advantage of this approach is the accurate determination of changes in the amplitude of distance components under turnover conditions where a subjective determination of the background in DeerAnalysis can distort these changes. This software is available from Matlab Central (http://www.mathworks.com/matlabcentral/fileexchange/46729-deera2012-zip).

## Additional files

### Major datasets

The following previously published datasets were used:

HohlM, BriandC, GrutterMG, SeegerMA, 2012, Crystal structure of a heterodimeric ABC transporter in its inward-facing conformation, http://www.pdb.org/pdb/explore/explore.do?structureId=3QF4, Publicly available at RCSB Protein Data Bank.

WardA, ReyesCL, YuJ, RothCB, ChangG, 2007, Crystal Structure of MsbA from Salmonella typhimurium with AMPPNP, higher resolution form, http://www.pdb.org/pdb/explore/explore.do?structureId=3B60, Publicly available at RCSB Protein Data Bank.
